# German Screen for Child Anxiety Related Emotional Disorders (SCARED): Reliability, Validity, and Cross-Informant Agreement in a Clinical Sample

**DOI:** 10.1186/1753-2000-4-19

**Published:** 2010-06-30

**Authors:** Katharina Weitkamp, Georg Romer, Sandra Rosenthal, Silke Wiegand-Grefe, Judith Daniels

**Affiliations:** 1 Department of Child and Adolescent Psychiatry, University Medical Centre Hamburg-Eppendorf, Martinistraße 52, 20246 Hamburg, Germany; 2 Department of Neuropsychiatry, University of Western Ontario, London, Canada

## Abstract

**Background:**

The psychometric properties and cross-informant agreement of a German translation of the Screen for Child Anxiety Related Emotional Disorders (SCARED) were assessed in a clinical sample

**Methods:**

102 children and adolescents in outpatient psychotherapy and their parents filled out the SCARED and Youth Self Report/Child Behaviour Checklist (YSR/CBCL).

**Results:**

The German SCARED showed good internal consistency for both parent and self-report version, and proved to be convergently and discriminantly valid when compared with YSR/CBCL scales. Cross-informant agreement was moderate with children reporting both a larger number as well as higher severity of anxiety symptoms than their parents.

**Conclusion:**

In conclusion, the German SCARED is a valid and reliable anxiety scale and may be used in a clinical setting

## Background

Anxiety disorders are a widespread phenomenon in children and adolescents [1-3]. Due to the covert nature of the symptoms, these disorders often remain underdiagnosed and untreated. Ravens-Sieberer and her colleagues reported that less than half of the children affected by severe anxiety symptoms received treatment at the time of the assessment, although the anxiety pathology considerably affected their well-being and functioning [3]. The undertreatment of anxiety disorders may be due to the fact that children and adolescents with internalizing symptoms do not exhibit interpersonal behaviour problems that would exact a thorough diagnostic. Considering the fact that, in some children, anxiety pathologies persist into adulthood or act as a risk factor for the development of other psychiatric disorders later in life [4,5], the lack of treatment for most of the children and adolescents with anxiety symptoms demands attention.

Therefore, an economic and easily administered screening instrument for anxiety disorders can serve as an important first step towards the identification of psychopathology and indicate the need of treatment in otherwise undiagnosed children and adolescents. To date, anxiety questionnaires exclusively assessing a specific diagnosis are prevalent in German-speaking countries, for example, screenings for social anxiety like the Social Phobia and Anxiety Inventory for Children (SPAIK; [6]) or specific phobias like the Phobia Questionnaire for Children and Adolescents (PHOKI; [7]). Other screening instruments such as the Children Anxiety Test (KAT-II [8]) or the Spence Children's Anxiety Scale (SCAS [9]) lack parallel parent versions.

The Screen for Child Anxiety Related Emotional Disorders (SCARED; [10]) is a broad screening instrument which offers a self- and a parent-report version. The instrument was developed on the basis of the DSM-IV classification of anxiety disorders, with five factors representing the main anxiety diagnoses. To date, the usability of the German SCARED parent version has not been tested. Furthermore it is unclear, whether there is sufficient agreement between parent and child reports and whether mother and father experience their child's symptoms in a similar way.

Analyses of cross-informant agreement in an English sample showed moderate correlations between parent- and self-report versions of the SCARED (r = .55 for total score; r = .40 to .58 for subscales; [11]). On average, child reports yielded higher scores than parent reports (total score: M = 18.12 vs. M = 14.43), which was mainly attributable to the responses on somatic/pain and separation anxiety subscales [11]. A review article analyzing general agreement between different informants reported frequent discrepancies in the ratings of emotional and behavioural problems in children (e. g. correlations about r = .20; [12]). In the review by Achenbach and colleagues, parents seemed to report higher levels of symptom severity. This could be attributable to the focus on disruptive behaviours which tend to be underreported by children and adolescents [12,13]. Cross-informant agreement between children and their parents thus seems to vary by disorder, with slightly better agreement in anxiety disorders, but certainly needs to be considered low overall. Previous investigations suggest that anxious children report a larger number of symptoms compared to their parents' accounts when describing the child [11,14]. This applies to most internalizing disorders due to the covert character of the symptoms. Therefore, child reports of anxiety symptoms are generally considered valid. However, in social phobia, the validity of child self-reports seems to be questionable, as socially phobic children tend to report less symptoms than their parents due to the fear of negative evaluation [15].

So far the usability of the German parent version of the SCARED has not been tested. Furthermore, the German SCARED has not been used in a clinical sample. The aim of this study was thus to test the feasibility and psychometric properties of the German SCARED in a clinical sample and to examine the cross-informant agreement between both parents and the child. Therefore the German SCARED was used with young patients enrolled in outpatient psychotherapy treatment as well as with their parents.

This article investigates (1) whether the German parent- and self-report versions of the SCARED prove reliable in a clinical sample, (2) whether convergent and discriminative validity of the questionnaire can be established, and (3) whether the cross-informant agreement of father-, mother- and self-report is satisfying.

## Methods

### Procedure

Data collection was carried out as part of a naturalistic effectiveness trial on child and adolescent psychotherapy in Northern Germany. The study has been approved by the ethics committee of the Hamburg Medical Association. 25 child and adolescent psychotherapists in private practices supported the study. 102 families with a child or adolescent enrolled in psychotherapeutic treatment and diagnosed with a psychiatric disorder participated in the study between September 2007 and August 2009. For children under the age of 11 years, only parent reports were collected. Patients from the age of 11 years (n = 61) were asked for their self-report. Since some of the administered instruments are only constructed and validated for children aged 11 years and older this age cut-off was chosen. In 14 cases adolescents did not consent to the inclusion of their parents into the study.

At the beginning of the outpatient therapy, patients and both parents (if available) were asked to participate by the therapist. Additionally, families received a letter informing them of the study and the later use of the collected data and signed an informed consent. The families then received questionnaires and instructions via mail. Children completed the child version of the SCARED (SCARED-C), and each parent completed the parent version of the SCARED (SCARED-P), separately. A pre-paid self-addressed envelope was included to facilitate participants' cooperation. Families who failed to return the questionnaires received two reminder letters after two and four weeks with backup questionnaires attached. Where possible, patients' diagnoses were established using the Schedule for Affective Disorders and Schizophrenia for School-Age Children-Present Episode (K-SADS-P; [16]). For this purpose, parents as well as patients aged eleven years and older were interviewed by a trained psychologist.

### Sample

The sample consisted of 102 children and adolescents, as well as their parents, attending psychoanalytical outpatient psychotherapy treatment. For patients under the age of 11, only parent-reports were obtained. Patients 11 years and older (n = 61) were asked for their self-report as well as reports from both parents. In 14 cases (14%), adolescents did not consent to the inclusion of their parents. 82 mothers and 57 fathers filled out questionnaires. In 50% of the cases (n = 51), reports from both parents were obtained. In the remaining cases, the questionnaire was completed only by the mother (n = 31, 30%) or only by the father (n = 6, 6%). For a sub-sample of n = 30 all three informant sources (father, mother, and patient reports) were available.

Patients age ranged between 6 and 18 years (mean = 12.5 years). About two thirds of the sample were female (n = 64; 63%). Most children were Caucasian (>95%). More than 41% came from divorced families. Approximately 53% of the parents reported having graduated from high-school, about 30% of the patients reported at least one parent holding a technical or university degree. For a subgroup of 30 patients, father, mother, and patient reports were available. This subsample (average age: 14.7; range: 11-18; n = 22 female, i. e. 73%) will hence be used for the assessment of cross-informant agreement. This subsample does not differ significantly in terms of age and gender from the other participants aged 11 years and older with only one or no parent report (age: *t *= .668, p > .05; gender: χ^2 ^= .642; p > .05).

All participants had a diagnosed mental disorder. Diagnosis was established either by K-SADS interview conducted by a trained psychologist (n = 74; 73%) or by therapist diagnosis (n = 28; 35%). Of the 74 patients who participated in the diagnostic interview, 33 had an anxiety disorder (45%). Eighteen children/adolescents suffered from posttraumatic stress disorders (PTSD, 24%), thirty one from an affective disorder (42%), and 19 from a disruptive disorder (26%). 15 suffered from other disorders (20%), mainly enuresis, encopresis and tics. The patients diagnosed with an anxiety disorders exhibited substantial comorbidity with other anxiety disorders (n = 8), depressive (n = 9) and disruptive disorders (n = 7), PTSD (n = 2), and other disorders (n = 5). Only four patients qualified exclusively for one anxiety disorder.

### Instruments

The instruments for the current study were taken from a broader assessment battery which was compiled for the evaluation study.

In the present study, the first statistically validated German translation of the SCARED was used (41 item version, [17], available from the authors). The items of the SCARED consist of short and simple statements in the first person or, for the parent version, of statements referring to the child. Each item is scored on a scale from 0 to 2, with 0 = 'not true or hardly ever true', 1 = 'sometimes true', and 2 = 'true or often true'. The five subscales are panic/somatic (13 items; e.g., "When I feel frightened, it is hard to breathe"); generalized anxiety (9 items, e.g., "I worry about things working out for me"); separation anxiety (8 items, e.g., "I get scared if I sleep away from home"); social phobia (7 items, e.g. "I feel nervous with people I don't know well"), and school phobia (4 items, e.g. "I get stomach aches at school"). By summing across relevant items, subscale scores and a total score can be obtained, with higher values indicating higher degrees of anxiety.

Psychometric properties of the English version are good, with an internal consistency of α = .90 [18]. For the 41-item version, Birmaher and his colleagues suggested a cut-off at 25 points for pathological anxiety [18]. The SCARED was successfully translated into a range of different languages such as Dutch [19], Italian [20], Spanish [21], and Chinese [22]. The Dutch SCARED proved feasible in a clinical setting [23] and proved reliable in differentiating anxiety from other affective disorders in clinically referred youths [24]. A recent study on the usability of a German SCARED translation showed promising results in a German community sample [25]. The self report scales showed good internal consistency (α = .91 for the total score, α = .66 to α = .81 for the subscales). Confirmatory factor analysis of the German version supported the intended 5-factor structure, although a subsequent exploratory factor analysis showed that a 4-factor structure is equally likely [17].

In addition to the SCARED, subjects were also administered the Child Behavior Checklist for parents (CBCL; [26]) or Youth Self Report for children and adolescents (YSR; [27]), respectively. The YSR/CBCL consists of 118 items on specific emotional and behavioural problems in childhood and adolescence. Parent- and self report version contain corresponding syndrome subscales: social withdrawal, somatic complaints, anxious/depressed, social problems, thought problems, attention problems, delinquent behaviour, and aggressive behaviour. An internalizing and an externalizing symptom score can be calculated from the corresponding syndrome scales. Each item stands for a specific problem behaviour and is rated on a 3-point scale from "not true = 0" to "very true or often true = 2". The reliability and validity of these widely used instruments have been examined in a number of studies [27].

### Analysis

Symptom scores of the SCARED and the CBCL/YSR were calculated according to the published instructions (i.e. tolerating a maximum of 10% missings; [10,18,26,27]). For the SCARED, missing values were replaced by the individual subscale mean. For the CBCL/YSR, scores could not be calculated for one mother (1.3%), two fathers (3.6%), and two patients (3.3%) due to too many missing values. Analyses of the psychometric properties of the SCARED were carried out for each informant individually (father, mother, and child/adolescent). Reliability of the SCARED was estimated using internal consistency, namely Cronbach's alpha, for the total score and each subscale. Pearson correlations were used to examine the convergent and discriminant validity as well as the cross-informant agreement. Mean values were compared using analysis of variance and GLM. Data were processed with SPSS 15.0 and the sample was checked for violation of assumptions. To ensure a sufficient sample size, a power analysis was conducted beforehand with GPower [28]. A power of 80% is seen as a standard in clinical studies [29]. Under the assumption of medium effects, an optimal sample size of n>21 results for correlations and of n>24 for analysis of variance (ANOVA). Results and effect sizes are evaluated based on established conventions [29].

## Results

The means and standard deviations for the SCARED are presented in Table 1 for each informant separately. Across the different informants, symptoms on generalized anxiety and social phobia were most commonly reported. 47% of the children and adolescents scored in the clinical range on the SCARED (cut-off ≥ 25, [18]). If the same cut-off was applied for the parent reports, 22% of the mothers and 16% of the fathers rated their child in the clinical range. Differences in means across the informants are analyzed in detail in section 3.3.

**Table 1:** Mean scores for each perspective, correlations between the YSR and SCARED self-report, and cross-informant agreement

	Child	Mother	Father	Correlations with YSR						Cross-informant Agreement		
**SCARED**	**Mean (SD)**	**Mean (SD)**	**Mean (SD)**	**Total**	**Internal**	**External**	**Anxious/Depr**.	**Somatic**	**Soc. With-drawal**	**Father ↔ Mother**	**Child ↔ Mother**	**Father ↔ Child**

	**(n = 61)**	**(n = 79)**	**(n = 54)**	**(child perspective; n = 61)**						**(n = 51)**	**(n = 44)**	**(n = 31)**

Total	25.07 (13.91)	16.53 (10.99)	12.47 (10.41)	.67**	.78**	.13	.70**	.58**	.64**	.62**	.54**	.62**

Somatic/panic	5.94 (4.51)	2.50 (3.57)	1.24 (2.26)	.58**	.71**	.07	.61**	.68**	.50**	.16	.52**	.17

Generalized anxiety	8.75 (4.97)	5.33 (4.10)	3.92 (3.79)	.69**	.76**	.21	.78**	.46**	.57**	.61**	.48**	.58**

Separation anxiety	3.01 (2.90)	3.38 (3.48)	2.59 (3.31)	.62**	.66**	.20	.63**	.44**	.57**	.47**	.46*	.56**

Social phobia	5.54 (3.30)	4.02 (3.87)	3.86 (3.86)	.37**	.46**	.08	.36**	.25*	.56**	.69**	.48**	.58**

School phobia	1.87 (1.96)	1.30 (1.84)	0.89 (1.45)	.41**	.44**	.06	.36**	.43**	.36**	.67**	.57**	.74**

Note: ** p ≤ .01; * p ≤ .05 (one-sided)

### Reliability

The internal consistency was high for each informant of the SCARED (mother α = .89; father α = .93; patient rating α = .94). Satisfactory Cronbach's Alphas resulted for the subscales, varying between α = .72 and α = .89 for self-report; between α = .76 and α = .90 for mother-report, and between α = .78 and α = .92 for father-report.

### Validity

#### Convergent validity

In order to analyze the convergent validity, associations between the SCARED scales and the total scores, the internalizing syndrome scores, as well as the corresponding subscales from the YSR/CBCL (social withdrawal, anxiety/depressive and somatic pain) were identified via bivariate correlations for each informant individually. Table 1 displays the correlations between the SCARED and the YSR for the self-report version. The scales proved to be significantly correlated in the expected direction. Correlations were higher for the SCARED total score than for the subscales, except for generalized anxiety which correlated highest with the YSR total score (r = .69, p ≤ .001). For the subscales of the YSR, correlations were highest with the internalizing syndrome score and lowest for somatic complaints.

#### Discriminant validity

In order to assess the discriminant validity of the SCARED, bivariate correlations with the externalizing subscale (YSR) were analyzed. As expected, these correlations showed no significant relationship between the SCARED and externalizing symptoms (see Table 1). Correlations for parent versions were computed as well, but are omitted due to space restrictions (see Additional file 1: Table S1). However, the correlations were in a comparable range.

### Cross-Informant Agreement

To analyse cross-informant agreement, bivariate correlations between scores retrieved from the father, the mother and the self-report version of the SCARED were calculated separately for the total score and the SCARED subscales. Cross-informant agreement between family members was all in the moderate to high range with one exception (see Table 1). Agreement between father and child as well as father and mother was low for the "somatic/pain" subscale, especially in comparison to the substantial agreement between mother and child (father/child: r = .17, p ≤ .181 and father/mother: r = .16, p ≤ .135 versus mother/child: r = .52, p ≤ .001). Overall correlations between informants were higher for the corresponding scale than with other subscales (e.g. mother rating of generalized anxiety was higher with child's ratings of generalized anxiety and lower with other subscales of child's ratings). Again, the only exception being father's rating of somatic/panic symptoms, which correlated higher with mother's and child's ratings of school phobia (r = .43, p ≤ .001 and r = .67, p ≤ .001) than with their ratings of somatic/panic symptoms (r = .16 and r = .17). Interestingly, the agreement for father and patient was higher than the agreement between mother and patient for the total score, generalized and separation anxiety as well as social and school phobia.

**Figure 1:**
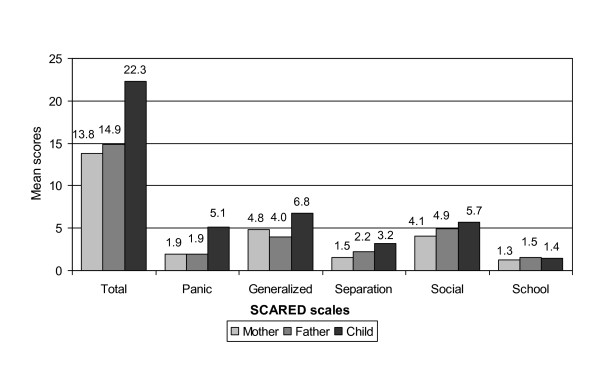
**Mean scores of the SCARED scales and total score for child/adolescent, mother, and father perspective (n = 28)**.

Considering previous research on cross-informant agreement, differences between parent- and self-reports in the degree of symptom report appear to be significant. Therefore, means were compared with an analysis of variance (GLM). Figure 1 shows the discrepancies between parent and child reports for the different SCARED subscales and the total score. Mostly, children and adolescents reported a larger number and/or more severe symptoms than father's and mother's ratings. For the total score, panic, separation, and generalized anxiety these differences are significant with large effect sizes (total: F = 8.056; p ≤ .001; eta^2 ^= .23; panic/somatic: F = 10.077; p ≤ .001; eta^2 ^= .258; generalized anxiety: F = 6.453; p ≤ .003; eta^2 ^= .193; separation anxiety: F = 5.346; p ≤ .008; eta^2 ^= .182). Social phobia and school phobia are reported to a comparable degree by the different sources of information with no significant differences (social phobia: F = 2.316; p ≤ .109; eta^2 ^= .082; school phobia: F = 0.241; p ≤ .787; eta^2 ^= .009).

Agreement with regard to whether the child fell in the clinical range (cut-off ≥ 25, [18]) of the SCARED was examined using cross tables. Parents agreed in 88% of the cases (Somer's d = .594, p ≤ .003). Parent-child agreement with regard to clinical status on the SCARED was 76% for father-child (Somer's d = .525, p ≤ .004) and 71% for mother-child accord (Somer's d = .418, p ≤ .007). In the case of non-agreement between a parent and the child report, almost always the child report was in the clinical range and the parent in the normal range (24% for father-child and 25% for mother-child non-agreement).

## Discussion

The main purpose of the article was to examine the reliability, validity, and cross-informant agreement of the German SCARED in a clinical sample.

The instrument yielded good psychometric properties in self- and parent report in a clinical sample. First of all, the administration of the SCARED was feasible in a sample of patients and their parents beginning psychotherapy treatment. Furthermore, internal consistencies were high for the total scores and satisfying for the scales. These results are consistent with the findings for the English version in a clinical sample and the German version in a non-clinical sample with Alphas for the total scores around α = .90 [18,25].

Convergent validity was supported by high correlations with internalizing CBCL/YSR symptom scores in the expected direction. Discriminant validity was supported by correlative independence from externalizing scores (CBCL/YSR). These findings mirror the results of Essau and her colleagues in the community sample with moderate to high correlations with internalizing and total scores of the YSR [25]. However, discriminant validity seemed to be lower in the community sample with moderate correlations of the SCARED scales with externalizing YSR scores [25].

Cross-informant agreement was in a moderate range and comparable to agreement scores for the English SCARED. The study by Wren and colleagues (2004) reports parent child agreement to average at r = .55 for the total score, while our study yielded correlations of r = .50 for father-patient agreement and r = .51 for the mother-patient agreement. Previous studies have generally found low to moderate parent-child agreement [10,12,18,30,31]. Compared to these studies, cross-informant agreement of the German SCARED can be considered being in the upper range in this sample. Previous studies did not differentiate between father and mother perspective. In our sample, the cross-informant agreement differs considerably by proxy and symptom group. While father and child agreement was higher for generalized and separation anxiety as well as social and school phobia, father's ratings of panic and somatic symptoms seemed to assess not panic but rather something similar to school phobia symptoms.

Although cross-informant agreement was acceptable in terms of correlational patterns, we also found strong differences for symptom severity and number of symptoms reported. Children and adolescents themselves tended to report more symptoms with higher degrees of intensity than their fathers and mothers. These findings are again consistent with research on the English version and other studies on informant agreement for anxiety ratings [11,15].

Interestingly, excess symptom reporting by children/adolescents did not occur for school and social phobia. This result replicates findings by DiBartolo and her colleagues [15] indicating that school and social phobia were underreported compared to reports of other anxiety symptoms which were related to the child's concern regarding positive self-presentation. It is possible that for self-representation concerns, these symptoms were played down. Therefore relying on child information alone for these subscales could lead to less valid assessments. Parents underreporting of somatic/panic, and generalized anxiety symptoms on the one hand, and that children refuse (and/or deny) symptoms of school and social phobia on the other hand, stress the importance of obtaining different perspectives in the diagnostic process of children and adolescents.

There are a number of potential limitations to this study. First, the sample size was relatively small. For this reason subgroups of the sample could not be contrasted with each other (e. g. different answering patterns for age and gender). Due to the sample size, optimal cut-off points for parents could not be determined. However, power analyses with GPower yielded satisfying power (>80%) for the current calculations. Secondly, it would be of interest, whether the SCARED is suitable to differentiate between anxiety and depressive symptoms. However, the ability of the instrument to discriminate between these disorders could not be tested adequately, as comorbidity with affective disorders was too high. As typical for clinical samples [2,32], about 20% of the children/adolescents with an anxiety disorder were also diagnosed with a comorbid affective disorder. Furthermore, future investigations could analyse a clinical group of only adolescents with anxiety disorders and then compare the subgroups of anxiety with the SCARED sub-scales, to test the ability of the SCARED to differentiate between anxiety disorders. Finally, we had no information on parents' diagnostic status, which has been found to influence their reporting of their children's problems [13].

Our study has a number of advantages as well. One advantage was the inclusion of father and mother perspective. Thus, the feasibility of the German parent-report version could be analyzed for both parents. A reliable and valid parent-report is necessary especially in younger children to complement self-reports with parent ratings for valid information. Furthermore, as the sample consisted of children and adolescents beginning outpatient psychotherapy treatment, this study was suitable to test the feasibility of the administration of the German SCARED in a clinical sample. The families seemed to be quite representative of the German population in terms of parents' education. 30% of the parents held a university or technical degree compared to about 24% in the general population [33]

A clinical implication derived from our data is that integrating both parent and child/adolescent perspectives should become the standard procedure in screening for anxiety disorders. Discrepancies in the degree of symptom reports between parents and children call for further research on individual cut-off-scores for the different perspectives.

In summary, good psychometric properties - comparable to the established English SCARED version - suggest the successful translation of the SCARED into the German language. Overall, the findings stress that the SCARED is a feasible, reliable, and valid screening instrument for parents and children/adolescents, and thus support the application of the German SCARED in clinical and research settings.

## Competing interests

The authors declare that they have no competing interests.

## Authors' contributions

KW has been responsible for the data analysis and the writing of the manuscript. GR, SWG and JD designed and coordinated the study, supervised the data analysis and the writing process. SR has been responsible for the coordination of the study. All authors have read and approved the final manuscript.

## Supplementary Material

Additional file 1 **Correlations between the CBCL and SCARED for mother and father report**. Click here for file
