# Supplementary material for: German Screen for Child Anxiety Related Emotional Disorders (SCARED): Reliability, Validity, and Cross-Informant Agreement in a Clinical Sample
Source: Child Adolesc Psychiatry Ment Health. 2010 Jun 30;4:19. doi: 10.1186/1753-2000-4-19 (PMC2912250; doi:10.1186/1753-2000-4-19)
Supplement: Additional file 1 — Correlations between the CBCL and SCARED for mother and father report. [file 1753-2000-4-19-S1.DOC]

Additional file 1: Table S1 - Correlations between the CBCL and SCARED for mother and father report

|  | **Mother report (n=80)** | | | | | | **Father report (n=55)** | | | | | |
| --- | --- | --- | --- | --- | --- | --- | --- | --- | --- | --- | --- | --- |
| **SCARED** | **Total** | **Internal** | **External** | **Anxious/ Depr.** | **Somatic** | **Soc. With-drawal** | **Total** | **Internal** | **External** | **Anxious/ Depr.** | **Somatic** | **Soc. With-drawal** |
| Total | .51** | .62** | .12 | .60** | .40** | .38** | .65** | .78** | .22 | .77** | .38** | .59** |
| Somatic/panic | .25* | .35** | -.02 | .34** | .43** | .12 | .40** | .56** | .07 | .64** | .40** | .28* |
| Generalized anxiety | .53** | .61** | .22* | .70** | .34** | .27** | .72** | .69** | .41** | .80** | .27* | .41** |
| Separation anxiety | .45** | .33** | .22* | .35** | .17 | .10 | .51** | .56** | .15 | .60** | .30* | .38** |
| Social phobia | .12 | .33** | -.19* | .22* | -.06 | .49** | .31* | .50** | -.00 | .33** | .22 | .62** |
| School phobia | .38** | .45** | .17 | .32** | .60** | .20* | .37** | .64** | -.04 | .51** | .60** | .53** |

Note: ** p≤.01; * p≤.05 (one-sided)
